# 3D holotomographic monitoring of Ca^++^ dynamics during ionophore-induced *Neospora caninum* tachyzoite egress from primary bovine host endothelial cells

**DOI:** 10.1007/s00436-021-07260-2

**Published:** 2021-08-13

**Authors:** C. Larrazabal, C. Hermosilla, A. Taubert, I. Conejeros

**Affiliations:** Institute of Parasitology, Biomedical Research Center Seltersberg, Justus Liebig University Giessen, Schubertstr., 81, 35392 Giessen, Germany

**Keywords:** *Neospora caninum*, Egress, A23187, 3D microscopy, Holotomography, Cattle

## Abstract

*Neospora caninum* represents an obligate intracellular parasite that belongs to the phylum Apicomplexa and is a major abortive agent in bovines. During merogony, *N. caninum* tachyzoites invade and proliferate in host cells in vivo, including endothelial cells of lymphatic and blood vessels. The egress at the end of the lytic cycle is tightly regulated in apicomplexans. Evidence in *Toxoplasma gondii* shows that Ca^++^ signalling governs tachyzoite egress. Much less is known on egress mechanisms of *N. caninum*. Here, we show, using 3D live cell holotomographic microscopy in fluo-4 AM-loaded *N. caninum*-infected BUVEC, that treatments with the calcium ionophore A23187 at 24- and 42-h post-infection (h p. i.) induced a fast and sustained increase in Ca^++^ signals in parallel to tachyzoite egress. A23187 treatments exclusively triggered tachyzoite release at 42-h p. i. but failed to do so at 24-h p. i. indicating a role for meront maturation in calcium-induced tachyzoite egress. Overall, we show that live cell 3D holotomographic analysis in combination with epifluorescence is a suitable tool to study calcium dynamics related to coccidian egress or other important cell functions.

**Supplementary Information:**

The online version contains supplementary material available at 10.1007/s00436-021-07260-2.

## Introduction

*Neospora caninum* is a heteroxenic apicomplexan parasite which belongs to the coccidian cyst-forming Sarcocystidae family (Lindsay and Dubey 2020). This family includes other important representatives of the phylum Apicomplexa for domestic, wild animal and human health, such as *Toxoplasma gondii*, *Sarcocystis suihominis* or *Besnoitia besnoiti*. *N. caninum* is a primary cause of abortion in bovines as well as other small ruminant species and has a substantial economic impact on livestock industry (Reichel et al. 2013). Since *N. caninum* is an obligate intracellular parasite, asexual formation of offspring stages (tachyzoites) strictly occurs in and depends on nucleated host cells. In vivo, *N. caninum* preferentially infects endothelium but may also invade other nucleated cell types, thereby provoking major changes in host cell functions (Horcajo et al. 2017; Velásquez et al. 2019; Regidor-Cerrillo et al. 2020) and finally leading to cell lysis. In this context, primary bovine endothelial cells have been proven as suitable for *N. caninum* in vitro replication, allowing high proliferation rate of tachyzoites (Taubert et al. 2006a, 2006b).

The successful replication cycle of tachyzoites starts with an active cell invasion, continues with intracellular parasite replication after parasitophorous vacuole (PV) formation and ends with active tachyzoite release which occurs after achieving full maturation (Behrendt et al. 2008). All these intracellular steps are critical for rapid parasite development (Black and Boothroyd 2000). From a physiological perspective, intracellular Ca^++^ acts as coupling factor or essential second messenger for a variety of cellular functions (Carafoli and Krebs 2016). Overall, Ca^++^-related studies on the closely related coccidian parasite *T. gondii* have shown that increase in intracellular Ca^++^ concentration is pivotal for adequate tachyzoite motility, invasion (Mondragon and Frixione 1996) and egress (Endo et al. 1982). The egress process allows tachyzoites to disseminate within the infected organism, thereby influencing the outcome of disease. However, and despite the well-described role of Ca^++^ in coccidian biology at functional level, the precise mechanisms underlying parasite egress are not fully understood (Caldas and de Souza 2018). In this context, egress-related studies on *T. gondii* have consistently demonstrated that treatments with calcium ionophores, such A23187 or ionomycin, induce an early egress of tachyzoites in a Ca^++^-dependent manner (Endo et al. 1982; Black et al. 2000; Caldas et al. 2007; Behrendt et al. 2008). Moreover, Ca^++^-driven egress is a general mechanism, since not only other chemicals including ethanol (Arrizabalaga and Boothroyd 2004), DTT (Stommel et al. 1997) and nigericine (Fruth and Arrizabalaga 2007), but also physiological signals, such as nitric oxide (Yan et al. 2015) or interferon gamma (IFN-γ) (Niedelman et al. 2013), can evoke *T. gondii* egress by modulating calcium dynamics. Interestingly, in case of the coccidian parasite *Eimeria bovis*, treatments with A23187 failed to induce egress of merozoite I stages from macromeronts, but promoted a fast exit of sporozoites from recently invaded endothelial cells (Behrendt et al. 2008), suggesting not only parasite species but also stage-specific egress mechanisms in apicomplexan parasites.

Calcium flux imaging is a classical approach to study cellular Ca^++^ homeostasis (Russell 2011). However, conventional microscopy-based approaches have rather been problematic for characterizing the role of intracellular calcium dynamics in host-coccidian parasites interactions (Lovett and Sibley 2003) since microscopic techniques are often limited to the post-experimental merge of signals coming from the phase (PH) or differential interphase contrast (DIC). Alternative approaches include the use of genetically modified parasites or suitable dyes for live cell imaging (Frigault et al. 2009). However, in case of these approaches, the difficulties of working with genetically modified organisms in addition to fluorescence-associated challenges like quenching or phototoxicity must be solved (Brown 2007). In this context, the recently developed holotomographic microscopy allows non-phototoxic and high-resolution imaging in live cells (Sandoz et al. 2019). The digital holotomographic reconstruction is based on the refractive index (RI) of the different cell structures, thereby enhancing the information possible to obtain in cell systems. In line, holotomographic acquisition permits a 3D reconstruction of the RI-based tomogram, allowing not only the generation of high-resolution images, but also bringing novel information regarding the spatial distribution of cellular structures (Sandoz et al. 2019). In addition, one of the key advantages is that RI-based registries can be directly merged with fluorescence with a suitable frame rate, allowing direct analysis of host-parasite interactions. The aim of this work was to study the role of calcium influx in *N. caninum* tachyzoite egress in bovine endothelial primary cells, using a combined approach of 3D holotomographic microscopy and calcium probe fluo-4 AM-mediated fluorescence analysis.

## Material and methods

### Host cell culture

Primary bovine umbilical vein endothelial cells (BUVEC) were isolated as described previously (Taubert et al. 2006a). BUVEC were cultured at 37 °C and 5% CO_2_ atmosphere in modified ECGM (modECGM) medium, by diluting ECGM medium (Promocell) with M199 (Sigma-Aldrich) at a ratio of 1:3, supplemented with 500 U/mL penicillin (Sigma-Aldrich), 50 μg/mL streptomycin (Sigma-Aldrich) and 5% FCS (foetal calf serum; Biochrom). Only BUVEC monolayers of less than three passages were used in this study.

### Parasites and treatments

*Neospora caninum* (strain NC-1) was maintained in vitro as described elsewhere (Taubert et al. 2006a, 2006b) by culturing it for several passages in permanent African green monkey kidney epithelial cells (MARC145) in DMEM (Sigma-Aldrich), supplemented with 500 U/mL penicillin, 50 μg/mL streptomycin and 5% FCS. Cells were cultured at 37 °C and 5% CO_2_ atmosphere. Vital tachyzoites were collected from supernatants of infected cells by a centrifugation step (800 × *g*; 5 min) and suspended in modECGM for further experiments. For parasite infection experiments, BUVEC were seeded in 35-mm tissue culture μ-dishes (Ibidi®) and cultured (37 °C, 5 % CO_2_) until confluence. Vital *N. caninum* tachyzoites (MOI 3:1) were added to BUVEC cultures for 4 h for infection. Thereafter, non-invaded or dead tachyzoites were removed by a complete medium change.

### Live cell 3D holotomographic microscopy and image analysis

3D holotomographic images and videos were obtained for *N. caninum*-infected host cells at 24- and 42-h p. i. using a 3D Cell Explorer-fluo microscope (Nanolive®) equipped with 60× magnification (*λ* = 520 nm, sample exposure 0.2 mW/mm^2^ and a depth of field of 30 μm) and a fluorescence unit (CoolLED pE-300ultra). Each experiment was performed independently three times (i.e. newly seeded BUVEC and new infections each time). Images were acquired every 6 s in both refractive index (RI) and fluorescence channels. The raw data were analysed using STEVE software (Nanolive®) to obtain refractive index-based z-stacks. Digital staining of subcellular structures was performed based on generated RI data.

### Tracking of intracellular Ca^++^ fluxes

Intracellular Ca^++^ fluxes were visualized by the Ca^++^-sensitive dye fluo-4 AM (Invitrogen) following the manufacturer’s recommendations. Briefly, host cells were incubated for 30 min in fluo-4 AM (2.5-μM final concentration) at 37 °C and supplemented with pluronic acid (Invitrogen) in a dye/pluronic acid ratio of 1:1. Non-incorporated dye was removed by washing in sterile PBS and adding fresh modECGM. Calcium influx induction was induced by calcium ionophore A23187 treatments (15 μM, Sigma-Aldrich).

Post-processing analysis was performed by the use of Image J v1.52p software (Schneider et al. 2012). Z Project plugin was applied to holotomographic z-stacks with the average intensity of the images as projection output. For calcium flux measurements over time, defined areas surrounding resting host cells were defined as regions of interest (ROI) (Silvestre-Roig et al. 2019). Thereafter, the multi-measurement tool (roiManager “Multi Measure”) was used to quantify the mean grey value as indicator of fluorescence intensity over time. Finally, for better visualization of Ca^++^ flux, fluorescence images were displayed in pseudo-colours, using fire lookup tables as described elsewhere (Ardiel et al. 2017; Liu and Baraban 2019; Wakida et al. 2020).

### Assessment of ionophore-induced tachyzoite egress

To estimate the impact of A23187 treatments, quantification of egressed *N. caninum* tachyzoites was performed at 24- and 42-h p. i. Briefly, at 24- and 42-h p. i., the number of meronts in *N. caninum*-infected BUVEC was determined (time 0). Then, the *N. caninum*-infected BUVEC cell monolayer was incubated with A23187 (15 μM) for 10 min and the number of meronts that released tachyzoites was determined by comparison with the image obtained at time 0. The result was expressed as percentage of meronts showing tachyzoite release, defining as the 100% the number of meronts with tachyzoites at the beginning of the experiment. At least 15 meronts per experimental condition were analysed.

### Statistical analysis

The statistical analyses were performed by GraphPad® Prism software (version 8.4.3). Ca^++^ fluxes were normalized as percentage of the maximal response in order to compare kinetic differences among experimental conditions. Global comparisons of Ca^++^ flux magnitudes were assessed by area under the curve (AUC) analysis at 1200-s post-stimulation. Data description was carried out by presenting arithmetic mean ± standard deviation (SD). In addition, the non-parametric statistical Kruskal-Wallis was applied to compare three or more experimental conditions. Whenever global comparison by Kruskal-Wallis test indicated significance, post hoc multiple comparison tests were carried out by Dunn tests to compare test with control (non-infected) conditions. Outcomes of statistical tests were considered to indicate significant differences when *p* ≤ 0.05 (significance level).

## Results

### Cellular Ca^++^ signals mainly originated from intracellular *N. caninum* meronts

**Figure 1.**
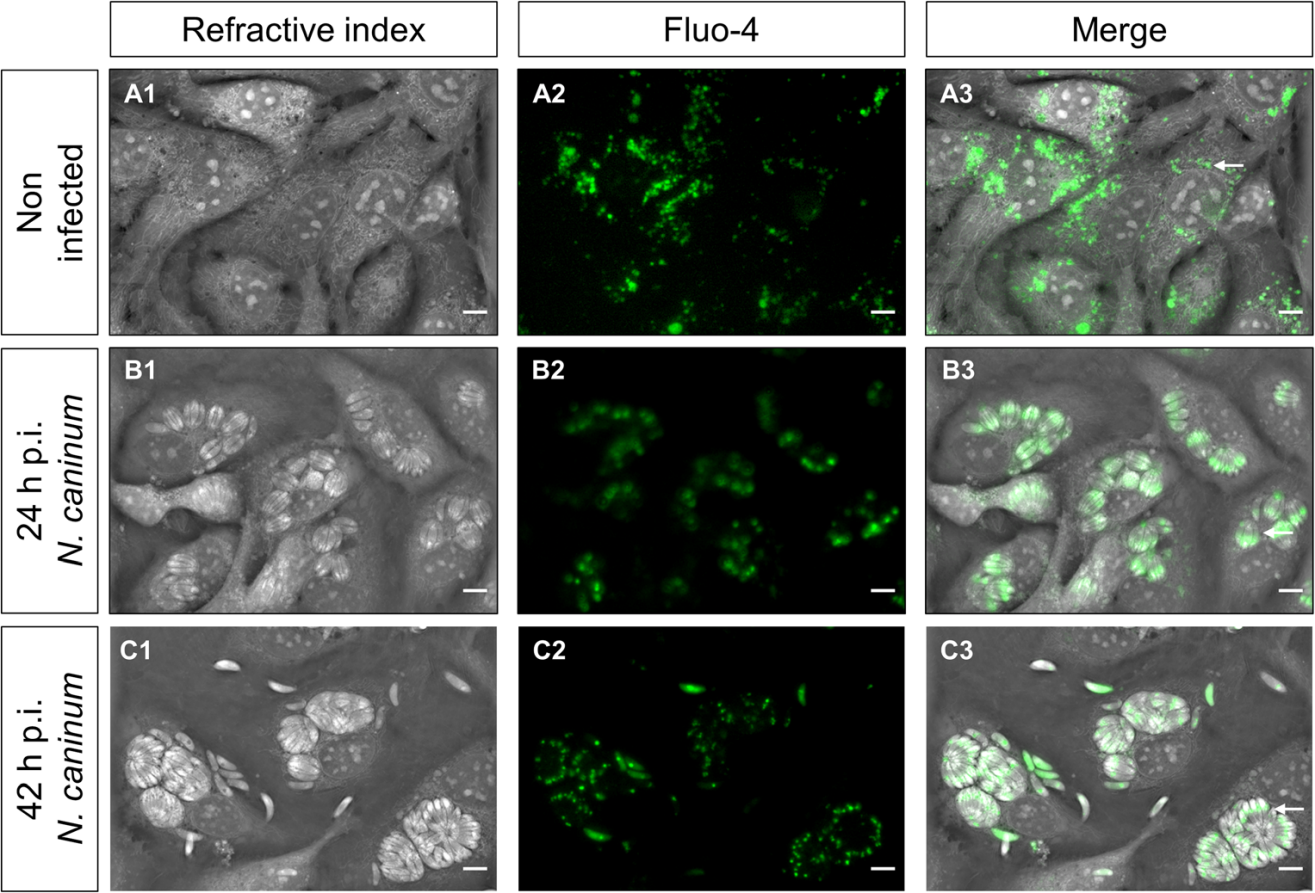
Calcium distribution in *N. caninum*-infected BUVEC determined by 3D tomographic microscopy. Refractive index (A1, B1 and C1) and fluorescent signal-based images (A2, B2, C2) of fluo-4-loaded BUVEC were obtained at 24- and 42-h p. i. Non-infected BUVEC were used as controls. Images exemplary illustrate calcium-derived signals (green fluorescence) of non-infected (**A**) and *N. caninum*-infected BUVEC at 24- (**B**) and 42-h p. i. (**C**). The white arrow in (A3) highlights perinuclear vesicle distribution and subapical calcium accumulation in intracellular tachyzoites (B3-C3). The full registry in video format can be found in the supplementary material. Size scale bars correspond to 5 μm

To characterize the localization of Ca^++^ signals within host cells, we performed a fluorescence and 3D holotomographic microscopy-based approach in *N. caninum*-infected (24- and 42-h p. i.) and non-infected BUVEC. Therefore, BUVEC were loaded with the Ca^++^-sensitive fluorescent dye fluo-4. As exemplary illustrated in Fig. 1, we observed a vesicle-like pattern of Ca^++^ accumulation in the perinuclear area of the cytoplasm in non-infected cells (Fig. 1 A3 arrow). Moreover, at 24- and 42-h p. i., *N. caninum*-infected BUVEC revealed a marked signal accumulation within meronts, specifically associated to the perinuclear area of tachyzoites (Fig. 1 B3; B4 arrow). No major differences in Ca^++^ signal pattern of infected cells were observed between 24- and 42-h p. i.

### A23187 treatments induced a fast and sustained Ca^++^ entry in *N. caninum*-infected host cells

**Figure 2.**
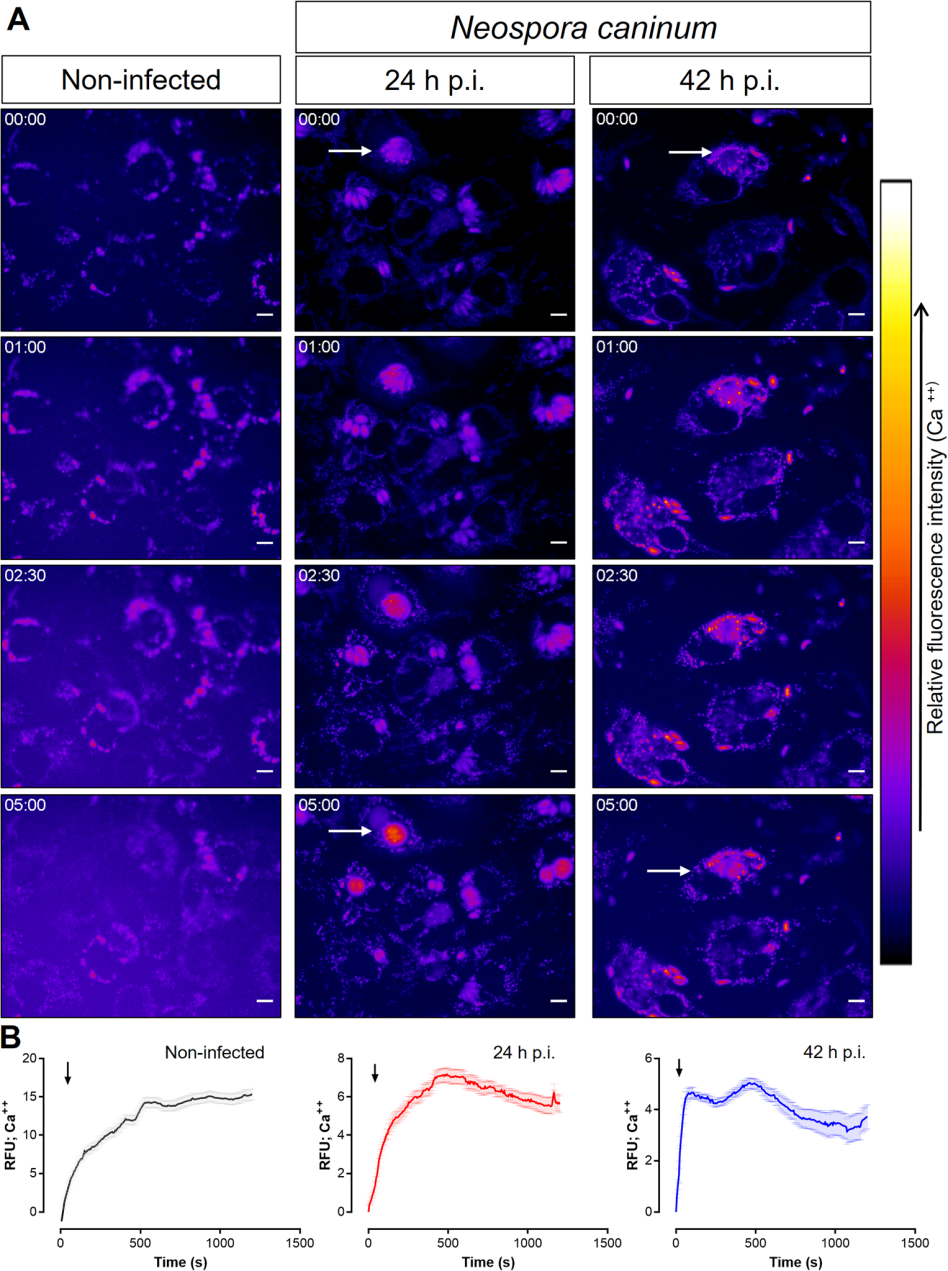
A23187 treatments induce a rapid and sustained calcium flux in non-infected and *N. caninum*-infected BUVEC. Fluorescence-derived images and measurements on calcium fluxes induced by A23187. **A** The images illustrate the changes in cellular calcium (pseudo-colour) dynamics in non-infected and *N. caninum*-infected BUVEC. Arrows highlight Ca^++^-driven signal accumulation in meronts over time. **B** Image-derived fluorescence intensity measurements over time in A23187-treated cells. Error bars express standard error of at least 5 cells. **C** The full registry in video format and the AUC analyses of image-derived fluorescence can be found in the supplementary material. Size scale bars correspond to 5 μm

Given that ionophore A23187 is capable of permeating cytoplasmic membranes for Ca^++^ ions, its use in cell systems allows to determine the role of Ca^++^ entry. As depicted in Fig. 2A, A23187 treatments of BUVEC induced an increase in Ca^++^-related fluorescent signals over time. Moreover, a time-dependent redistribution of Ca^++^-driven signals was detected at 24- and 42-h p. i. in *N. caninum*-infected host cells (Supplementary Videos 1 and 2). In addition, no evident cytotoxic effect like cell lysis or membrane protrusions during the first 5 min was observed. When referring to subcellular areas, an enhancement in fluorescent signals over time was detected within *N. caninum* meronts (Fig. 2A, arrows). Furthermore, quantitative image-based fluorescence registries showed an A23187-induced fast and sustained increase of Ca^++^-related fluorescence signals in both *N. caninum*-infected host cells and non-infected controls (Fig. 2B). Here, the current data showed that A23187 stimulation increased the fluo-4-driven signals reaching a peak after 500-s post-exposure in the non-infected and 24-h p. i.-infected cells, which was faster reached (60-s post-exposure) in 42-h p. i.-infected cells. This was followed by a plateau phase being in general sustained over time in all experimental conditions: non-infected cells, 24-h p. i. and 42-h p. i. Differences in the dynamics of the calcium flux were observed (Fig. 2B) whilst the magnitude of the Ca^++^ influx (defined as the AUC of the registries) was not significantly affected by the different experimental conditions (*p* = 0.47) (Fig. Sup. 1).

### Ionophore-induced *N*. *caninum* egress is tachyzoite maturation-dependent

**Figure 3.**
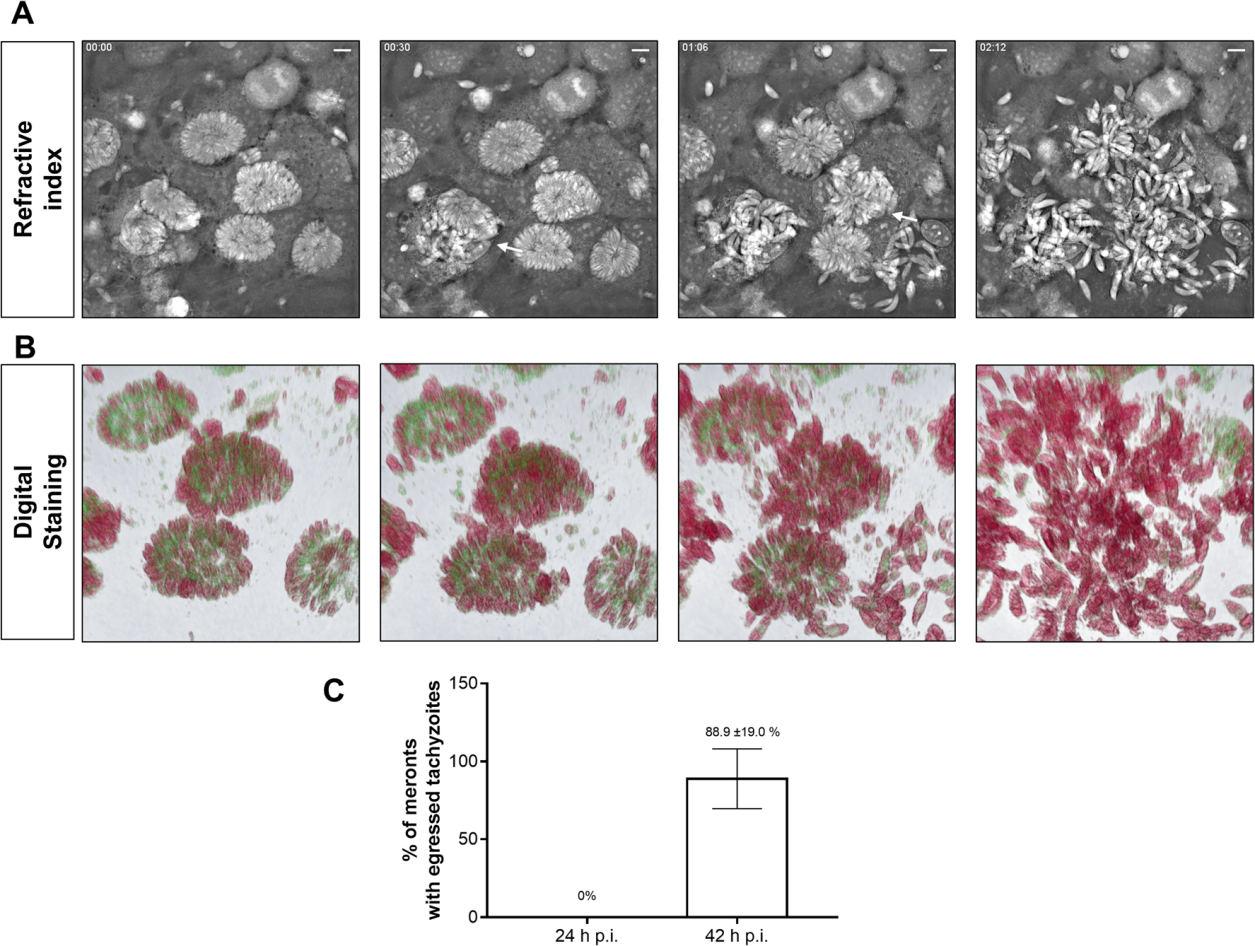
Calcium influx induced by A23187 treatment triggers a fast tachyzoite egress from *N. caninum*-infected BUVEC at 42-h p. i. Holotomographic images show a fast tachyzoite egress induced by A23187 treatments from *N. caninum*-infected BUVEC. Refractive index (**A**) or digital staining (**B**). Arrows highlight an early rosette disassembly and massive egress of motile tachyzoites from mature meronts. (**C**) Bar graph illustrating the mean of the percentage of meronts releasing tachyzoites after 10-min post-A23187 treatments ± standard deviation. The full registry in video format can be found in the supplementary material. Size scale bars correspond to 5 μm

During *N. caninum* meront development, tachyzoites divide several times within the infected cell and finally egress in a Ca^++^-dependent process. To evaluate the dependence of egress on meront maturation, the number of meronts showing tachyzoite release was determined at 24- and 42-h p. i. The data revealed that at 24-h p. i., no *N. caninum* meronts (0%) showed tachyzoite egress upon ionophore treatment. On the contrary, at 42-h p. i., 88.9 ±19.0 % of meronts released tachyzoites after 10 min of treatment (Fig. 3C). Interestingly, holotomographic videos revealed that at 24-h p. i., intracellular tachyzoites showed little or no motility within meronts after A23187 exposure (Supplementary Video 1). In contrast, at 42-h p. i., tachyzoite movements highly increased and calcium localization changed upon A23187 treatment finally leading to tachyzoites egress shortly after exposure (Fig. 3, Supplementary Video 2). These results were consistently illustrated by RI-based digital staining, showing rapid parasite rosette breakdown and hypermotility of intracellular tachyzoites within the PV (Fig. 3 Supplementary Videos 3–4).

## Discussion

Calcium flux imaging represents a suitable and reliable tool for the study of cellular Ca^++^ physiology (Russell 2011). Moreover, the use of microscopy-based approaches of host cell-parasite interaction studies allows to characterize the role of Ca^++^ fluxes in coccidian biology (Lovett and Sibley 2003). Despite that, using live cell fluorescence microscopy, the characterization of host cell-parasite interactions is usually limited to overlay approaches using light microscopic images (phase or differential interphase contrast). These limitations can be overcome by the use of genetically modified fluorescent parasites or dyes suitable for live cell imaging (Frigault et al. 2009). However, the complexity of genetic manipulations and multi-channel fluorescence-associated problematics, such as quenching or phototoxicity (Brown 2007), generates a complex experimental set-up for studying intracellular parasites. Within this scenario, the current report shows for the first time that changes in Ca^++^ fluorescence signal intensities can be documented by RI-based illustration using a holotomographic microscope. Here, this technique allowed us to simultaneously monitor 3D holotomographic and epifluorescent signals to characterize the dynamics of intracellular Ca^++^ fluxes induced by A23187 treatments in vitro. In addition, the use of fluo-4 AM as Ca^++^-sensitive dye delivered sensitive and specific signals with reduced interference from other bivalent cations (Gee et al. 2000). In detail, we here found that Ca^++^-mediated fluorescent signals in non-infected endothelial control cells mainly originated from a perinuclear position, showing a vesicle-like pattern in the cytoplasmic region. This finding is in line with previous reports on bovine aortic endothelial cells and human umbilical endothelial cells (Chang et al. 2001; Son et al. 2010).

Cytoplasmic Ca^++^ distribution processes are highly conserved among eukaryotic cells, where endoplasmic reticulum (ER), mitochondria and Golgi are considered Ca^++^-rich organelles in resting cells (Russell 2011; Carafoli and Krebs 2016). The current study of apicomplexan-infected host cells proved fluorescence signals mainly to be located within *N. caninum* meronts, most possibly due to a redistribution of host cellular cytosolic Ca^++^. This observation is in agreement with previous reports on *T. gondii-*infected cells, where the Ca^++^ concentration was significantly higher in the PV than in the cytosolic area of human epidermoid carcinoma epithelial cells (24-h p. i., Pingret et al. 1996), indicating that the PV represents a specialized calcium-rich subcellular compartment. In detail, we here found that fluo-4-based signals predominantly translocated into the subapical region of intracellular *N. caninum* tachyzoites. In line with other eukaryotic organisms, tachyzoites in principle possess above described Ca^++^-rich organelles (Lourido and Moreno 2015) but they additionally own other Ca^++^ stores, the so-called acidocalcisomes, as described for *T. gondii* and *Plasmodium berghei* (Moreno and Zhong 1996; Marchesini et al. 2000).

The calcium ionophore A23187 is a mobile ion carrier that forms stable complexes with cations, such as Ca^++^ (Pressman 1976). As already reported in the past, treatments with this compound effectively triggered Ca^++^ fluxes and other apicomplexan tachyzoite egresses (Endo et al. 1982; Black and Boothroyd 2000; Behrendt et al. 2008). As here illustrated, A23187 treatments effectively induced increases in Ca^++^-derived fluorescent signals over time in *N. caninum*-infected and control BUVEC. Considering cellular compartments, Ca^++^ signals mainly accumulated in vesicle-like structures within the cytoplasmic compartment of non-infected host endothelial cells. More interesting, in *N. caninum*-infected BUVEC, cytoplasmic signals were quickly redistributed to tachyzoite stages within meronts, thereby demonstrating a rise in tachyzoite intracellular Ca^++^ levels; nevertheless, the possibility that this Ca^++^ signal enhancement is a consequence of a Ca^++^ flux from *N. caninum* tachyzoite intracellular stores originated by the egress induction should not be excluded. This represents the first report on Ca^++^ flux evaluation during *N. caninum* tachyzoite egress. Furthermore, quantitative analyses on fluorescence intensities using defined ROIs demonstrated that A23187 treatments provoked a fast calcium flux in 42-h p. i. *N. caninum*-infected cells and with a similar kinetic in non-infected and 24-h p. i.-infected cells. The latter was followed by a sustained plateau phase, most probably reflecting the stabilization of extra- and intracellular Ca^++^ concentrations. Interestingly, during Ca^++^ induction at 42-h p. i., slight changes in Ca^++^ flux over time were observed; however, this can be consequence of the movement of tachyzoites during the ionophore-induced egress, rather than reflecting an alternative kinetic profile, which, in general, matches with a receptor-independent mechanism and seems consequence of channel formation in cytoplasmic membranes (Tang et al. 2007).

Mechanisms of coccidian egress have extensively been studied in *T. gondii* (Caldas and de Souza 2018). In contrast, much less data are available on other closely related apicomplexan parasites, such as *N. caninum*. So far, a pivotal role of Ca^++^-sensitive mechanisms has been proposed previously. As such, treatments with DTT and thiazolides derivatives induced a BAPTA-sensitive tachyzoite egress from *N. caninum*-infected human foreskin fibroblasts (evaluated 30-min post-induction at 48-h p. i.; Esposito et al. 2007). Likewise, 10-μM A23187 treatments evoked egress in infected BUVEC 10-min post-treatment at 60-h p. i. (Behrendt et al. 2008). Unfortunately, and despite the relevance of these works, the mere use of phase contrast microscopy hindered precise characterization of calcium influx signals in tachyzoite egress in living cells and only allowed for limited data interpretation. Using the current technique, *N. caninum* tachyzoites showed highly motile activities and egressed after 30 s of ionophore exposition at 42-h p. i. but failed to do so at 24-h p. i. In line, low intracellular tachyzoite motility was observed in reaction to A23187 exposition at 24-h p. i. Since *N. caninum*-infected BUVEC showed a similar calcium dynamic profile at 24- and 42-h p. i., we speculate that egress rather depends on meront maturity than on the mode of calcium influx. This is in agreement with previous observations showing that the maturity of meront-contained *T. gondii* and *N. caninum* tachyzoites significantly influenced A23187-triggered egress in BUVEC (Behrendt et al. 2008). Interestingly, *T. gondii* egress seems to involve an additional pathway, namely an active process from the tachyzoite side (Caldas and de Souza 2018). Specifically, studies on *T. gondii* demonstrated the participation of a perforin-like protein (tgPLP-1) and a lecitin:cholesterol acyltransferase (tgLCAT), which are secreted from the parasite micronemes and dense granules, respectively, and which both proved pivotal for PV disintegration and tachyzoite release (Kafsack et al. 2009; Pszenny et al. 2016). In line, electron microscopy-based analyses confirmed that tubular network disintegration within the PV is an early event during *T. gondii* egress (Caldas et al. 2010). Considering this, motility seems crucial for ionophore-induced *T. gondii* egress (Lavine and Arrizabalaga 2008). Consequently, forming tachyzoites within immature *N. caninum* meronts may indeed be fixed in a tubular network within the PV, thereby failing to egress upon ionophore treatments. Comparative studies on *T. gondii* and *N. caninum* have shown that A23187-induced egress is highly influenced by infection kinetic and maturity of intracellular meront stages (Behrendt et al. 2008), thereby indicating that ionophore-induced early egress is highly affected by parasite-specific characteristics. However, and despite the well-documented role of Ca^++^ in *T. gondii*-induced cellular egress, the correlation of calcium flux and coccidian egress has not sufficiently been characterized via microscopy-based approaches, yet.

In summary, current data showed that *N. caninum* tachyzoites egress is a Ca^*++*^-sensitive mechanism. Also, differences in tachyzoite egress behaviour depending on the time post-infection and resulting maturation status were here documented. These differences were not necessarily dependent on the Ca^*++*^ influx and further investigations are needed to better understand this cellular process. From a methodological perspective, the current study shows the usefulness of novel live cell 3D holotomography in combination with the use of fluo-4 AM as a suitable tool for calcium-related studies dealing with coccidian egress and development in host cells.

## Supplementary Information

ESM 1 (PNG 19783 kb)

High Resolution Image (TIF 883 kb)

ESM 2 (MP4 11616 kb)

ESM 3 (MP4 12315 kb)

ESM 4 (MP4 2261 kb)

ESM 5 (MP4 5414 kb)

## Data Availability

All data are included in the manuscript.
