# Supplementary figures and images for: 3D holotomographic monitoring of Ca++ dynamics during ionophore-induced Neospora caninum tachyzoite egress from primary bovine host endothelial cells
Source: Parasitol Res. 2021 Aug 13;121(4):1169–77. doi: 10.1007/s00436-021-07260-2 (PMC8986705; doi:10.1007/s00436-021-07260-2)

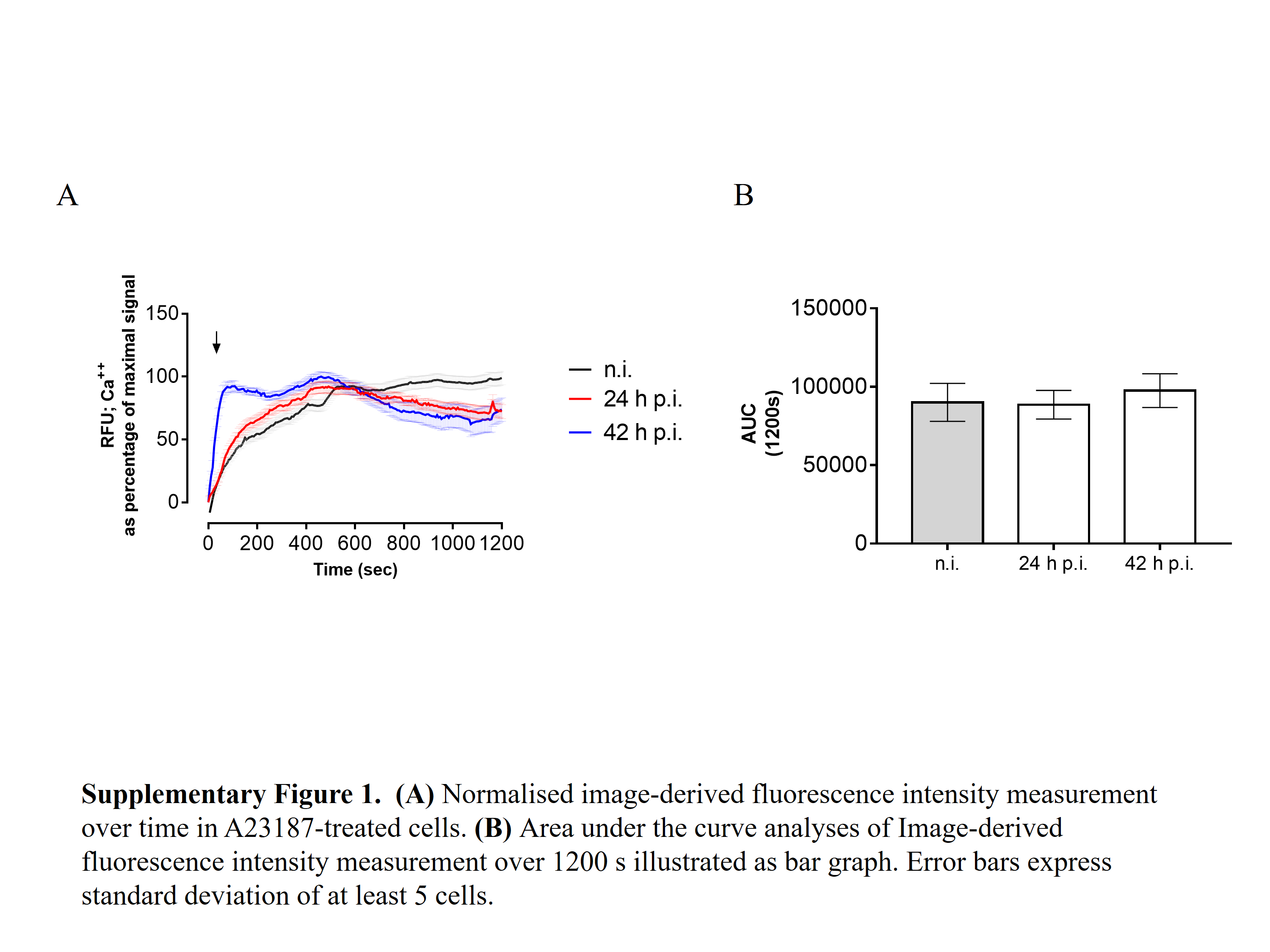

Supplement: Supplementary file 1 — (PNG 19783 kb) [file 436_2021_7260_Fig1_ESM.png]

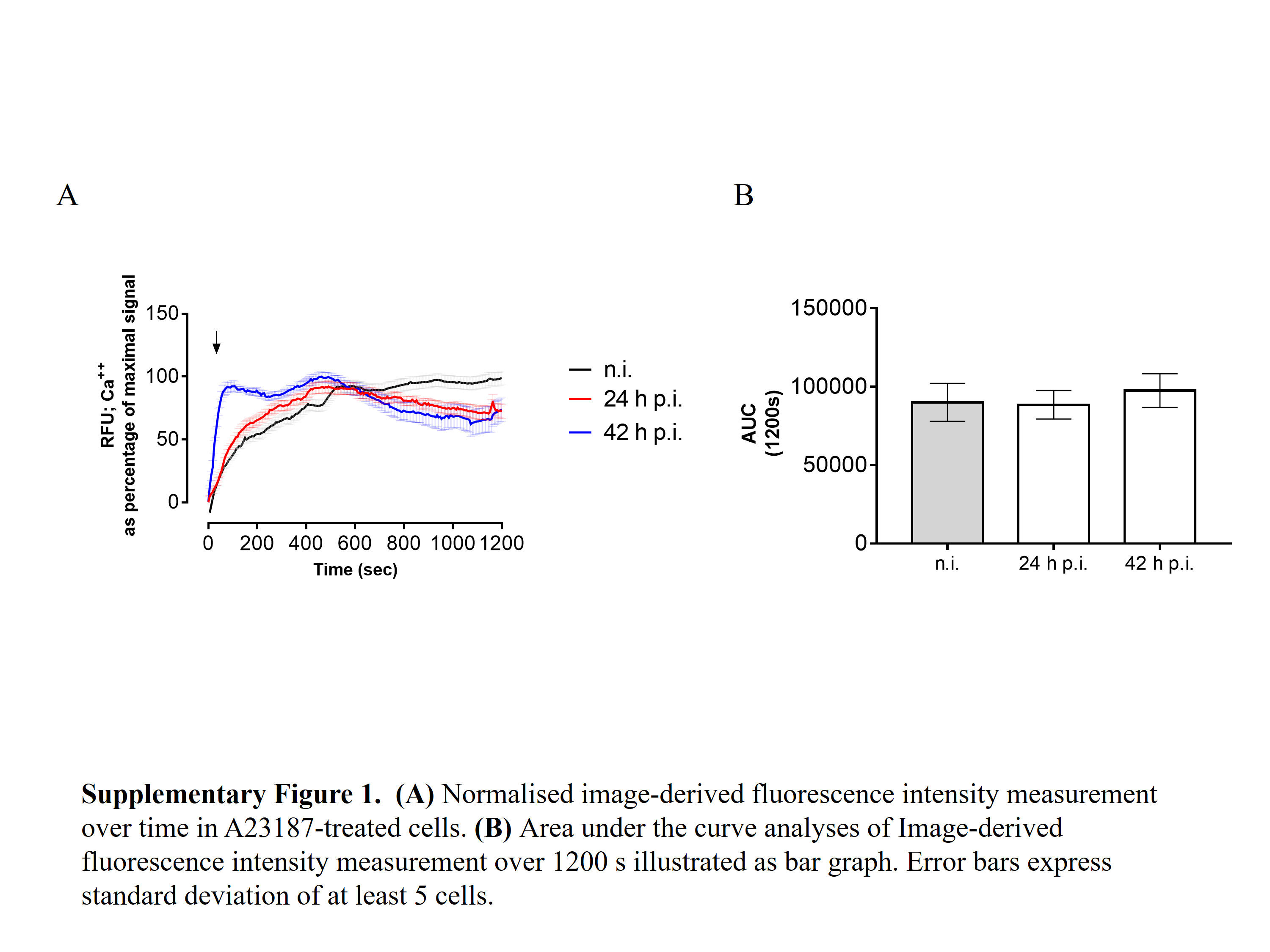

Supplement: Supplementary file 2 — High Resolution Image (TIF 883 kb) [file 436_2021_7260_MOESM1_ESM.tif]
